# A New Approach to Single‐Step Fabrication of TiO_*x*_‐CeO_*x*_ Nanoparticles

**DOI:** 10.1002/smsc.202400305

**Published:** 2024-09-09

**Authors:** Marie Elis, Tim Tjardts, Josiah Ngenev Shondo, Ainura Aliyeva, Alexander Vahl, Ulrich Schürmann, Thomas Strunskus, Franz Faupel, Cenk Aktas, Lorenz Kienle, Salih Veziroglu

**Affiliations:** ^1^ Chair for Synthesis and Real Structure Department of Materials Science Faculty of Engineering Kiel University Kaiserstraße 2 24143 Kiel Germany; ^2^ Chair for Multicomponent Materials Department of Materials Science Faculty of Engineering Kiel University Kaiserstraße 2 24143 Kiel Germany; ^3^ INP Greifswald Leibniz Institute for Plasma Science and Technology Felix‐Hausdorff‐Str. 2 17489 Greifswald Germany; ^4^ Kiel Nano Surface and Interface Science KiNSIS Kiel University Christian Albrechts‐Platz 4 24118 Kiel Germany; ^5^ Department of Orthodontics University Hospital of Schleswig‐Holstein (UKSH), Kiel University Arnold‐Heller‐Straße 3 Kiel 24105 Germany

**Keywords:** defect formation, deposition rate, gas aggregation cluster source, mixed metal oxides, TiO_2_‐CeO_2_ nanoparticles

## Abstract

Mixed metal oxide (MMO) nanoparticles (NPs) are hybrids consisting of two or more nanoscale metal oxides. Advantages of MMO NPs over single metal oxides include improved catalytic activity, enhanced electrical and magnetic properties, and increased thermal stability due to the synergy of the different oxide components. This study presents a novel fabrication route for TiO_2_‐CeO_2_ NPs enriched with oxygen vacancies using a Haberland‐type gas aggregation cluster source. The NPs, deposited from different segmented Ti/Ce targets under varying O_2_ addition, were examined with respect to final composition, morphology, and Ti, Ce surface oxidation states. Particle formation mechanisms are proposed for the observed morphologies. Additionally, available O_2_ during deposition and its impact on the formation of defective sites were investigated. Defective sites in TiO_2_‐CeO_2_ NPs were analyzed using transfer to X‐ray photoelectron spectroscopy and transmission electron microscopy without contact to ambient oxygen. The incorporation of Ce to the target exhibits synergistic effects on the synthesis process. Segmented Ti/Ce targets enable the deposition of a broad range of mixed oxide NPs with diverse compositions and morphologies at considerably enhanced deposition rates, which is vital for practical applications. The presented fabrication approach is expected to be applicable for a broad variety of MMO NPs.

## Graphical abstract

A novel approach for the synthesis of TiO_*x*_‐CeO_*x*_ NPs based on a gas aggregation cluster source equipped with a single segmented target is presented. Analytical results from particles synthesized at different conditions reveal the highly defective nature of the particle surface as well as the presence of various morphologies. Particle formation mechanisms for different scenarios are proposed.

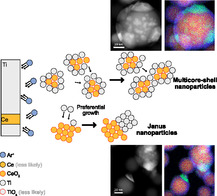

## 1 Introduction

Mixed metal oxide (MMO) nanoparticles (NPs) are hybrid materials composed of two or more metal oxides combined at the nanoscale. These NPs exhibit unique electrochemical, photocatalytic, and antibacterial properties.^[^ ^1^, ^2^, ^3^, ^4^ ^]^ MMO NPs offer several advantages over their single metal oxide components, including, for example, enhanced catalytic activity, improved electrical and magnetic properties, and increased energy storage capacity.^[^ ^5^, ^6^, ^7^ ^]^ Due to these exceptional properties, they find applications in various sectors, such as catalysis (as catalysts and catalyst supports), photocatalysis, environmental protection, energy storage and conversion, biomedical applications, and synthesis of advanced ceramic materials.^[^ ^2^, ^3^, ^7^, ^8^, ^9^ ^]^ The methods of fabricating MMO NPs influence the resulting properties and play a crucial role for their purity, morphology, and size distribution.^[^ ^10^, ^11^, ^12^, ^13^ ^]^ Optimizing these fabrication techniques is essential to fully harness the potential of MMO NPs in specific applications.^[^ ^14^, ^15^ ^]^

TiO_2_‐CeO_2_ is one of the commonly investigated MMOs for a variety of applications, including photocatalysis,^[^ ^16^, ^17^ ^]^ environmental catalysis,^[^ ^18^, ^19^, ^20^ ^]^ electrochemical sensors, and solar cells,^[^ ^21^ ^]^ as well as high refractive‐index optical films.^[^ ^22^ ^]^ The main reason why TiO_2_‐CeO_2_ is highly useful for various applications is due to its enhanced catalytic properties, which are a result of the synergistic effects between the two oxides. TiO_2_ is well‐known for its photocatalytic activity, while CeO_2_ is known for its oxygen storage capacity and redox properties. The coexistence of both Ce^4+^ and Ce^3+^ oxidation states is crucial for achieving high catalytic efficiency.^[^ ^23^, ^24^ ^]^ When combined, these two oxides create a material with improved reducibility, oxygen deficiency, acidity, and oxidation activity, making it highly effective for a wide range of catalytic applications.^[^ ^25^ ^]^ In general, TiO_2_‐CeO_2_ can be synthesized by a multitude of chemical synthesis routes such as the hydrothermal methods,^[^ ^20^ ^]^ sol‐gel methods,^[^ ^26^, ^27^ ^]^ and coprecipitation methods.^[^ ^28^ ^]^ However, chemical approaches often result in the formation of TiO_2_‐CeO_2_ nanocomposite with varying oxidation states of cerium (Ce^4+^ and Ce^3+^). The control of these oxidation states is a severe challenge in the case of chemical synthesis routes.^[^ ^25^, ^29^, ^30^ ^]^ Furthermore, these approaches often result in the formation of TiO_2_‐CeO_2_ nanocomposites with nonuniform dispersion of cerium ions, which can affect the catalytic activity and stability of the material.

In general, vacuum‐based deposition techniques offer several advantages over conventional wet chemical approaches for synthesizing thin films and nanocomposites.^[^ ^31^, ^32^ ^]^ These techniques provide improved film quality with fewer impurities due to the absence of atmospheric contamination and solvents.^[^ ^33^ ^]^ They also allow for precise control over film properties such as thickness, composition, and structure by manipulating vacuum conditions and deposition rate.^[^ ^33^ ^]^ Furthermore, vacuum‐based deposition can be used to deposit a wider range of materials that may react with the atmosphere.^[^ ^34^, ^35^ ^]^ Recently, we have used vacuum‐based DC magnetron sputter processes to achieve transparent TiO_2_‐CeO_2_ thin films with high purity and a defined TiO_2_‐CeO_2_ interface.^[^ ^36^ ^]^

Another notable vacuum‐based deposition technique is the sputter gas aggregation cluster source (GAS) approach.^[^ ^37^ ^]^ This approach involves the formation of NPs in a vacuum chamber by sputtering a target material in an inert gas atmosphere (with the potential to add reactive gas). The resulting NPs are then aggregated and deposited onto a substrate.^[^ ^38^, ^39^ ^]^ The GAS approach offers several benefits compared to conventional deposition processes.^[^ ^40^ ^]^ It enables the fabrication of high‐purity and well‐controlled nanocomposites with tailored properties.^[^ ^41^ ^]^ Additionally, the GAS approach allows for the production of NPs with specific shapes, sizes, and compositions.^[^ ^42^ ^]^ Moreover, using segmented metallic targets, gas phase synthesis of a broad range of mixed metal and metal oxide NPs is attainable.^[^ ^43^, ^44^, ^45^ ^]^ Depending on the material system, potential pathways to create MMO NPs either pursue in‐plasma addition of reactive oxygen gas or use post‐deposition oxidation. However, one major limitation of the GAS technique is the relatively low production rate of NPs, especially in the case of metal oxides.^[^ ^46^, ^47^ ^]^ While for common noble metal targets, such as Ag, Pt, or Au, a stable and continuous deposition is commonly obtained, more reactive metals such as Ti typically require meticulous control over the admixture of reactive gases in order to show considerable deposition rate.^[^ ^48^ ^]^ This imposes challenges for the scalability of the process for industrial applications. Moreover, the GAS approach can be highly sensitive to changes in operating conditions, which can affect the size and properties of the NPs produced.^[^ ^49^, ^50^ ^]^

In this study, we fabricated highly defective TiO_*x*_‐CeO_*x*_ NPs using a Haberland‐type GAS. The novelty of our approach is the combination of the MMOs via reactive magnetron sputtering with the technique of GAS. This enabled us to prepare nanosized mixed oxide particles directly from the sputter deposition process. The fabricated particles were deposited from a metallic composite Ti‐Ce target under different oxygen availability conditions in the chamber. We primarily investigated the effect of Ce content in the composite Ti‐Ce target on the final composition and type of the deposited TiO_*x*_‐CeO_*x*_ NPs and the impact of available O_2_ during the deposition process on the formation of defective sites in these NPs. Additionally, we explored how to properly investigate the defective sites in TiO_*x*_‐CeO_*x*_ NPs using direct vacuum transfer to an X‐ray photoelectron spectroscopy (XPS) system as well as transfer via an inert gas atmosphere to a transmission electron microscope (TEM). The role of Ce in the stability of the deposition process and the creation of defective sites is investigated, which is crucial for practical applications.

## 2 Results and Discussion

**Figure 1:**
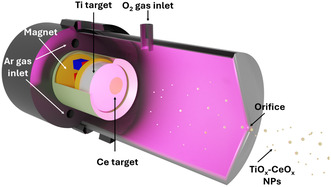
Schematic representation of the GAS setup with a segmented Ti‐Ce metallic target.

The sputter GAS process is a method for producing NPs by aggregating atoms and molecules that were released by magnetron sputtering in an aggregation vacuum chamber.^[^ ^51^, ^52^ ^]^ **Figure** **1** schematically shows a setup for producing metal NPs by the GAS process. Usually, GAS utilizes nonreactive sputter gases impinging a target that leads to the release of atoms and dimers (among other sputtered species) to the gas phase.^[^ ^53^, ^54^ ^]^ Then, stable nuclei within the aggregation chamber grow to NPs by the processes of coagulation, coalescence, and aggregation^[^ ^55^ ^]^ in a highly complex mechanism.^[^ ^56^, ^57^ ^]^ In GAS involving the reactive gas O_2_ in addition to a metal target and an inert sputter gas, the sputtered species interact with the reactive gas to form metal and oxygen‐containing molecules. These molecules have a much higher binding energy than pure metal dimers, which facilitates the formation of more stable dimers, intermediate compounds, and resulting nuclei compared to nonreactive approaches.^[^ ^48^, ^58^, ^59^ ^]^ In contrast, the reactive O_2_ can also cause poisoning of the target surface.^[^ ^60^ ^]^ This takes place in two ways: The first is gettering of the reactive gas at the target surface.^[^ ^61^, ^62^ ^]^ Here, a direct reaction with the target surface atoms takes place and produces dielectric compounds.^[^ ^60^ ^]^ In a second mechanism of redeposition, metal oxide species arrive on the target from the gas phase and are incorporated into the growing oxide.^[^ ^63^ ^]^ However, this deposition and poising process becomes highly complex when two metals, which have different oxygen affinities and sputter yields, are combined in a single composite target.

### 2.1 Formation of TiO_*x*_‐CeO_*x*_ Nanoparticles with Additional Oxygen

**Figure 2:**
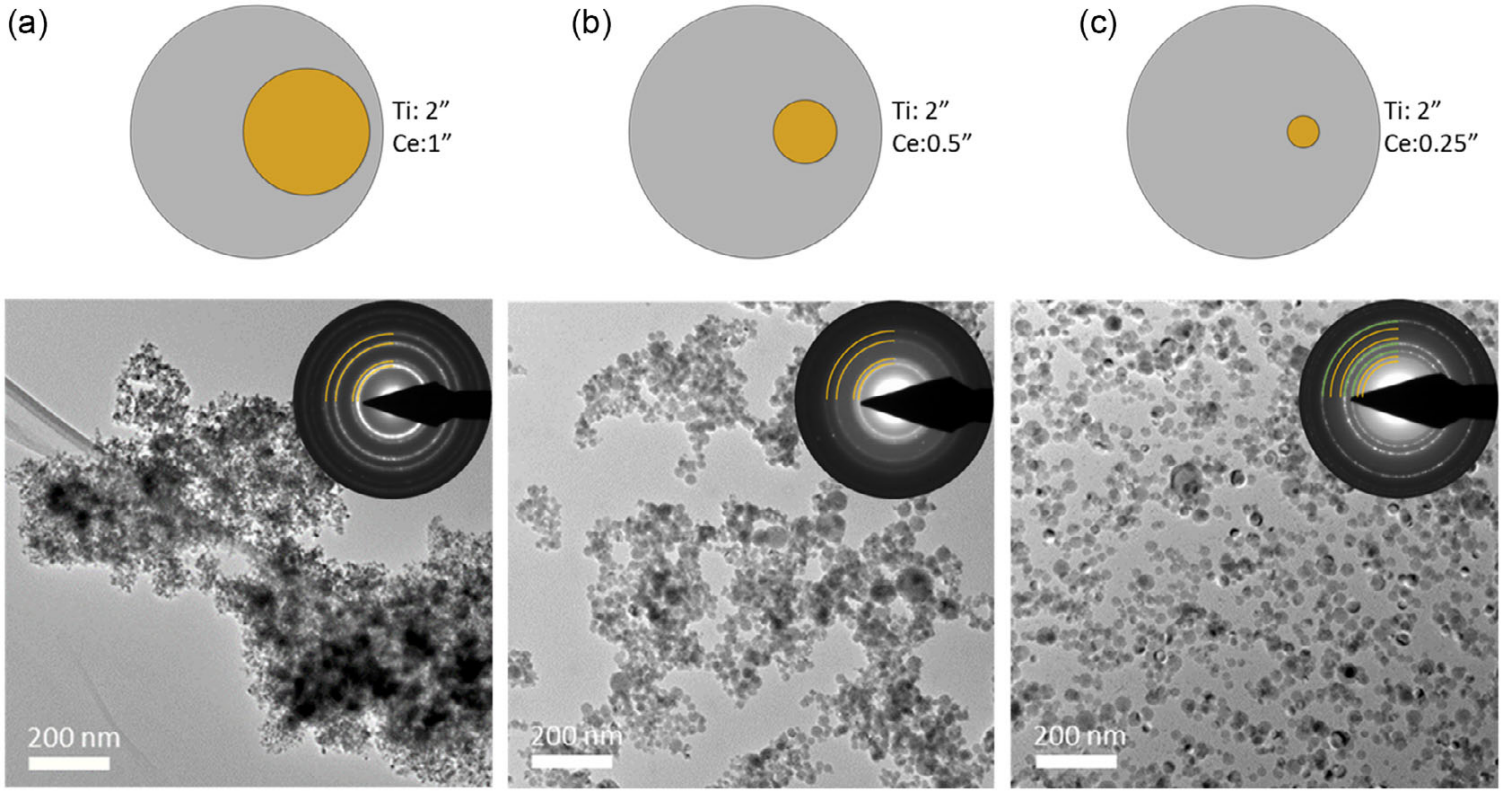
NPs deposited from targets with different sizes of Ce inlet targets at 50–70 W magnetron power, 30–50% duty cycle, 30 sccm Ar flow, and 0.015 sccm O_2_ flow. In the first row, sketches of the targets with a) 1”, b) 0.5”, and c) 0.25” Ce inlet targets are shown. Below, BF TEM images of the resulting particles are presented. SAED patterns of the respective samples are shown as an inset with references for CeO_2_ ((111), (002), (022), and (113) reflections from ICSD 621 710)^[^ ^86^ ^]^ marked in yellow and TiO ((111), (002), and (022) reflections from ICSD 77 692)^[^ ^87^ ^]^ marked in green.

To investigate the resulting change in NP composition, three different Ti/Ce segmented composite targets consisting of 2” Ti targets with Ce inlet targets of different sizes were employed for NP synthesis. Sketches of these 2” Ti targets with inlet target diameters of (a) 1”, (b) 0.5” (b), and (c) 0.25” are shown in the first row of **Figure** **2**. In the first set of experiments, we produced Ti/Ce MMO NPs with the addition of external oxygen as a reactive gas. From the bright‐field (BF) TEM images of the NPs presented in Figure 2 below the respective target sketches, no obvious changes in the appearance of the particles due to the change of target can be observed. The strong agglomeration of the NPs in the TEM image (Figure 2a) is not a result of the deposition process but of the scratch‐off procedure applied for TEM preparation. The NPs in TEM in Figure 2b were transferred via ultrasonication and drop casting, while the NPs in Figure 2c were deposited directly on TEM grids, leading to increased homogeneity in particle distribution on the substrate. The selected area electron diffraction (SAED) patterns provided as an inset in the BF TEM images depict reflections corresponding to cubic CeO_2_ (marked in yellow) for all three samples. The appearance of additional reflections corresponding to cubic TiO in the diffraction pattern of Figure 2c hints toward an increased concentration of Ti in the sample. These TiO reflections might be too low in intensity to be visible next to the high intensity of the CeO_2_ reflections in the cases of Figure 2a,b. In the case of Figure 2c, the intensity of TiO reflections is increased, indicating a higher Ti concentration, while the intensity of the CeO_2_ reflection is reduced. This is also supported by the energy‐dispersive X‐ray (EDX) spectra acquired in TEM from a large number of NPs. The ratio of Ti to Ce content of the samples varied strongly for the three target configurations. To estimate the areal contribution of the Ce target to the overall sputter erosion zone, a simple geometric construction was applied, as shown in Figure S1a, Supporting Information: The Ce target (green circle) is placed with an offset of 9 mm (black line) from the center of the Ti target (blue circle). The erosion zone (yellow area) was assumed to span from an erosion zone radius of 10.8–14.8 mm, based on photographic images captured after the prolonged operation of a representative target inside the GAS. To obtain the Ce/Ti erosion zone area fraction, the overlap between the Ce target and the erosion zone (orange area) was determined numerically. Figure S1b, Supporting Information, provides a plot of the Ce/Ti ratios measured by EDX over the calculated erosion zone area fractions, revealing an approximately linear relationship. For the 1” Ce inlet, the Ce/Ti ratio (Ce/Ti erosion zone area fraction is 0.16) was determined for several samples from different depositions to be ≈80/20. When reducing the size of the Ce inlet to half the diameter (0.5”) (Ce/Ti erosion zone area fraction is 0.75), the Ce/Ti ratio was changed to ≈50/50. The reduction to 0.25” (Ce/Ti erosion zone area fraction is 0.01) resulted in a Ce/Ti ratio of ≈10/90.

**Figure 3:**
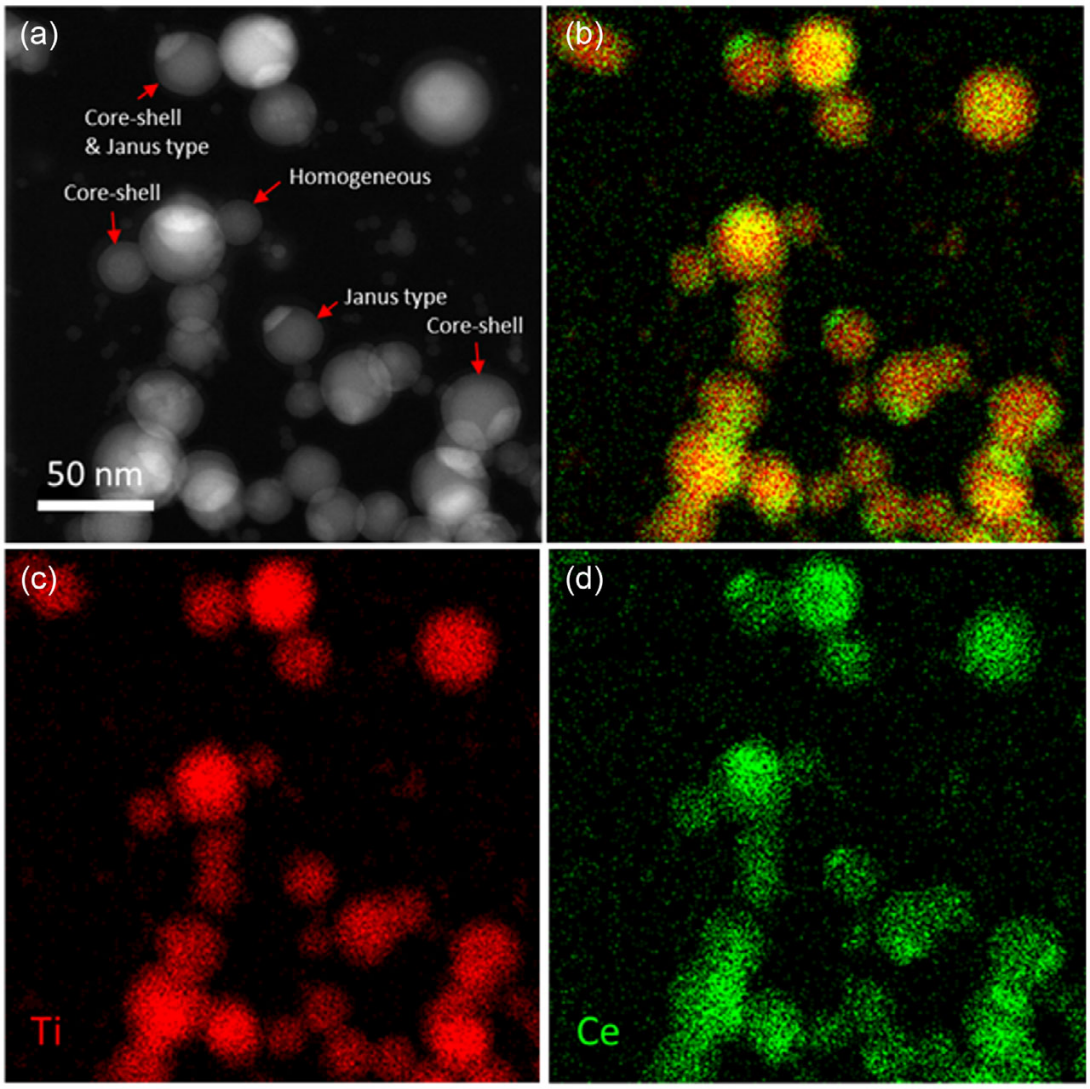
NPs deposited at 50 W, 30 sccm Ar, and 0.015 sccm O_2_ flow from a Ti target with a 0.25″ Ce inlet target. a) ADF STEM image with corresponding b) overlay EDX map, c) Ti elemental EDX map, and d) Ce elemental EDX map.

The high Z‐contrast of TiO_2_ and CeO_2_ makes annular dark‐field scanning TEM (ADF STEM) a well‐suited technique to observe the morphologies of the MMO NPs. In the image presented in **Figure** **3**a, core‐shell and Janus‐type as well as a few homogeneous NPs can be easily identified. The particles were synthesized from the Ti target with 0.25” Ce inlet and the addition of 0.015 sccm O_2_. The EDX maps in Figure 3b–d confirm that the bright “caps” of the particles in the ADF STEM image are Ce‐rich, while the darker major parts of the particles are Ti‐rich. The majority of particles shows this Janus‐type morphology of Ce “caps” and Ti “body.” Additionally, core‐shell morphology can be observed. The darker shell of the NPs results from a higher oxygen content in the shell. Figure S2, Supporting Information, provides an EDX line scan of an NP with Janus‐type as well as core‐shell morphology. From the intensity plot in Figure S2b, Supporting Information, it can be seen that the oxygen signal decays less strongly toward the edge of the particle than the Ti signal, indicating an increased oxygen concentration. The intermixing of the two oxides resulting from the synthesis in GAS appears to be low. Ce‐rich and Ti‐rich areas are clearly separated but still attached via an interface to form a single particle.

**Figure 4:**
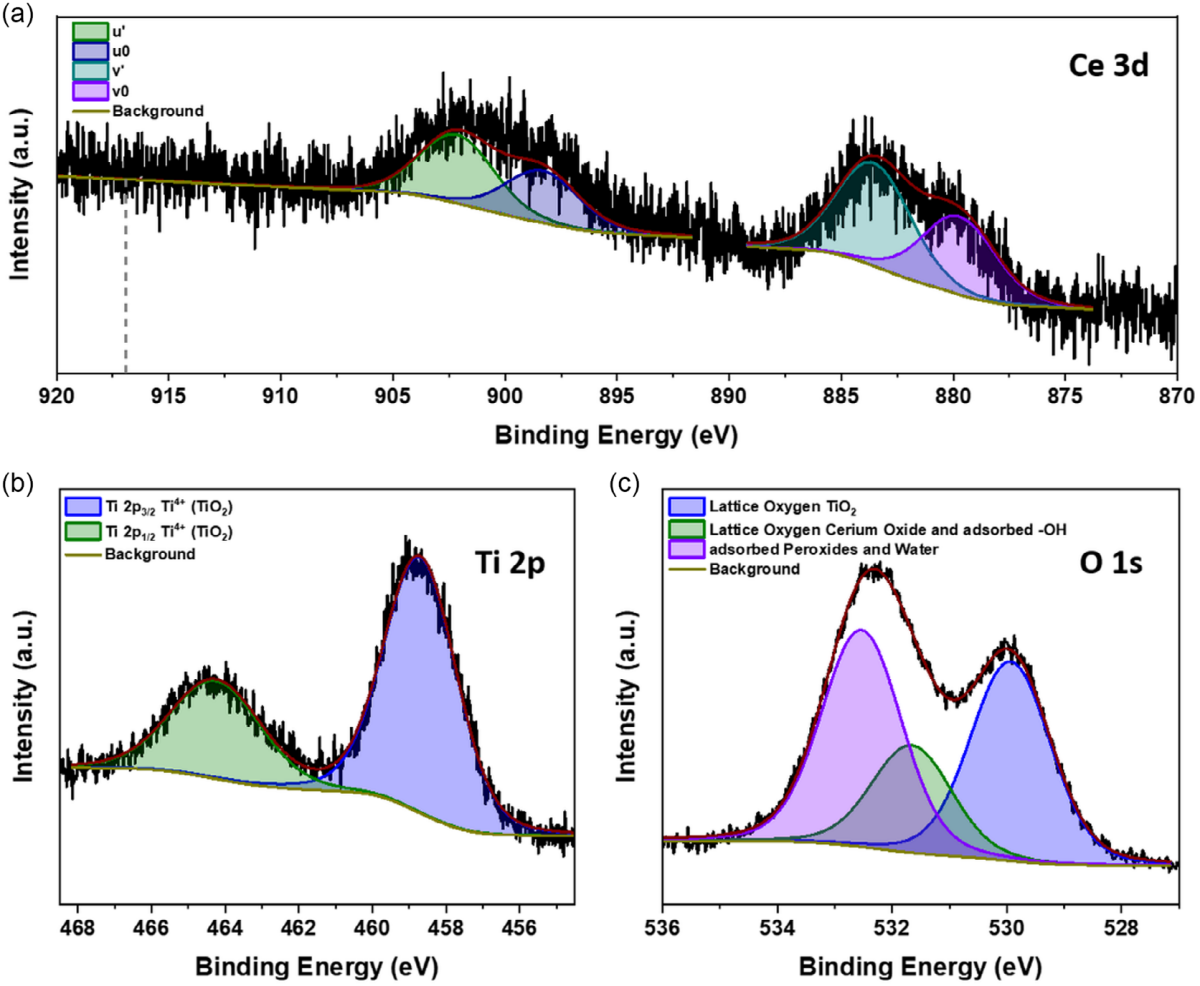
XPS core‐level spectrum of a sample produced with 0.015 sccm external O_2_ with respective fitting functions and background: a) Ce 3*d* with indicated binding energy position at 916.9 eV, b) Ti 2*p*, and c) O 1*s*.

The core‐shell morphology and the higher oxygen content in the shell motivate a detailed surface chemistry study via XPS. **Figure** **4**a depicts the respective Ce 3*d* spectrum. The spectrum shows two main features that correspond to the spin‐orbit splitting of the Ce 3*d* level. It splits into a high binding energy Ce 3*d*_3/2_ and a low binding energy Ce 3*d*_5/2_ component. Both of these components show two subpeaks. The subpeaks *u*′, *u*_0_ relate to Ce 3*d*_3/2_, and the subpeaks *v*′, *v*_0_ are associated with Ce 3*d*_5/2_.^[^ ^64^ ^]^ During the fitting of the subpeaks, we constrained the intensity ratio *I* (3*d*_5/2_)/*I* (3*d*_3/2_) to the reported value for the spin‐orbit splitting of 1.5 and set a fixed splitting of 18.6 eV according to Beche et al.^[^ ^64^ ^]^ The subpeaks u′ and v′ correspond to the final state of Ce 3*d* ^9^4*f* ^1^ O 2*p* ^6^, and the subpeaks u_0_ and v_0_ correspond to a respective shakedown feature with a final state of Ce 3*d* ^9^4*f* ^2^ O 2*p* ^5^. The spectrum strongly indicates the presence of Ce^3+^ at the NP's surface.^[^ ^64^, ^65^, ^66^, ^67^ ^]^ The absence of the Ce 3*d* ^9^4*f* ^0^ O 2*p* ^6^ final state‐specific component *u*‴ reported at around 916.9 eV^[^ ^64^ ^]^ is another strong indicator that Ce^4+^ is not a major part of the Ce at the surface. The cerium oxide at the surface most likely consists of Ce^3+^, which is in contrast to the overall CeO_2_ structure found by electron diffraction in Figure 2. However, we can interpret this result with the high reducibility of CeO_2_ due to the relatively low oxygen vacancy formation energy at the surface, which favors the surface reduction of Ce^4+^ to Ce^3+^.^[^ ^68^ ^]^ That means that the core of the cerium‐rich parts of the NPs consists of CeO_2_, but the surface contains many oxygen vacancy defects, and the electron diffraction pattern still indicates CeO_2_.

Figure 4b shows the corresponding Ti 2*p* spectrum. The Ti 2*p* signal displays two distinct peaks separated by ≈5.6 eV with an area ratio of ≈2.0. This is the characteristic spin‐orbit splitting into a high binding energy Ti 2*p*_1/2_ component with lower intensity and a low binding energy Ti 2*p*_3/2_ component with higher intensity.^[^ ^69^ ^]^ During the charge correction, we set the binding energy position of the Ti 2*p*_3/2_ component at 458.7 eV, which matches the TiO_2_ reference values according to the NIST database.^[^ ^70^ ^]^ Other XPS studies on cerium‐titanium mixed oxides also report the Ti in the highest possible oxidation state of Ti^4+^.^[^ ^64^, ^71^ ^]^ Therefore, it is highly likely that the Ti at the surface of the NPs prepared with external O_2_ is in a Ti^4+^ state. At first glance, this is in contrast to the cubic TiO we found in the NPs from the diffraction patterns in Figure 2. However, the increased oxygen content in the shell of the NPs found by the EDX line scans from Figure S2, Supporting Information, generally supports a full oxidation of Ti at the surface. Despite the observed fully oxidized Ti^4+^ at the surface, we also observed the reduced Ce^3+^ state, which is not the fully oxidized Ce oxidation state. We already emphasized the high reducibility of Ce^4+^ to Ce^3+^. However, here, we also have to consider the cerium oxide‐TiO_2_ interface that might support the presence of Ce^3+^. Johnston‐Peck et al. presented strong experimental and theoretical evidence that Ce^3+^ occurs at cerium oxide‐TiO_2_ interfaces due to the preference of oxygen vacancy formation,^[^ ^72^ ^]^ which other studies support as well.^[^ ^73^, ^74^ ^]^ Therefore, we can interpret the cerium oxide‐TiO_2_ interface as a possible contributor to the presence of the Ce^3+^ as well.

Figure 4c displays the corresponding O 1*s* spectrum. It shows three components that are associated with peroxides and adsorbed water (purple), adsorbed –OH groups and lattice oxygen in Ce_2_O_3_ (green), and lattice oxygen in TiO_2_ (blue). The lowest binding energy component at 529.9 eV corresponds to the reference values for lattice oxygen in TiO_2_.^[^ ^69^, ^70^ ^]^ The second lowest component at 531.7 eV relates to an overlap between lattice oxygen in Ce_2_O_3_^[^ ^66^, ^75^ ^]^ and adsorbed –OH groups. The adsorbed –OH groups are reported at approximately the same binding energies for Ti^[^ ^76^ ^]^ and Ce oxides.^[^ ^29^, ^75^ ^]^ The highest binding energy component at 532.5 eV most likely results from adsorbed oxygen in the form of peroxides and water.^[^ ^77^, ^78^ ^]^ The fact that this component is a major part of the O 1*s* peak indicates that a relatively large amount of peroxide is adsorbed on the sample surface. This matches the relatively high oxygen content at the surface found by the EDX line scans in Figure S2, Supporting Information. Since both the Ti 2*p* and the O 1*s* spectrum show strong indications for Ti^4+^, we take the difference Δ_O1*s*–Ti 2*p*,ref_ ≈ 71.2 eV in binding energy between their respective O 1*s* lattice oxygen and Ti 2*p*_3/2_ component as an indicator for Ti^4+^. This difference Δ_O1*s*–Ti 2*p*,ref_ will be used as a Ti^4+^ reference for the other XPS spectra we present in this study. We take this measure since C 1*s* charge correction does not have a perfect reliability^[^ ^79^ ^]^ and the physisorbed adventitious carbon on the surface might charge differently compared to the underlying surface. Therefore, we have applied a charge correction at the known Ti 2*p*_3/2_ component associated with Ti^4+^ and reported at 458.7 eV.^[^ ^76^ ^]^

As mentioned earlier, the fabrication of metal oxide NPs, such as TiO_2_, via the GAS process faces challenges due to the low deposition rate caused by target poisoning. This phenomenon significantly affects the formation of NPs. Typically, target poisoning refers to the formation of a stable oxide layer on the surface of the metal target during NP formation.^[^ ^80^, ^81^ ^]^ This oxide layer can hinder the sputtering of metal atoms, which are essential for forming metal oxide NPs.^[^ ^82^ ^]^ In the case of TiO_2_ NP formation by GAS, target poisoning can be controlled by adjusting the oxygen admixture in the working gas mixture. A low amount of O_2_ can promote the formation of TiO_2_ NPs, while a high amount of O_2_ can lead to target poisoning.^[^ ^48^, ^83^ ^]^ However, target poisoning is a highly dynamic process that depends on the balance between the formation and removal of the oxide layer. Consequently, it is challenging to control the continuous deposition properly, and the NP deposition typically stops when the quasi‐equilibrium is disrupted (the deposition rate of TiO_2_ NPs is around 0.2–0.55 Hz s^−1^ for ≈5 min). When Ce is used as an inlet target to form TiO_2_‐CeO_2_ NPs, the poisoning process might differ slightly from the general theory. The presence of Ce may influence the formation and time‐dependent evolution of the oxide layer on the target surface, potentially affecting the deposition rate and NP formation.

As it is well‐known, Ce has a much higher affinity for oxygen than Ti.^[^ ^84^ ^]^ During the deposition process, when external O_2_ is supplied to the chamber, most of the O_2_ molecules are primarily captured by the Ce target, which is leading to the initial nucleation of CeO_2_ NPs due to its high oxygen affinity. Basically, the top oxide layer (CeO_*x*_) on the metallic Ce target can be easily removed from the target surface by Ar bombardment due to low adhesion between the metallic and native oxide parts. This phenomenon facilitates the prefabrication of early CeO_2_ cluster nucleation in the deposition chamber. Consequently, the possibility of poisoning the Ti target is briefly reduced, promoting the growth of TiO_2_ clusters in the deposition chamber on top of the early CeO_2_ clusters. Stable and continuous NP deposition can be achieved when the overall process reaches an equilibrium between the metallic Ti and Ce targets and the formation of CeO_2_ and TiO_2_ NPs (the deposition rate of TiO_*x*_‐CeO_*x*_ NPs is around 1.44–1.73 Hz s^−1^ for ≈30 min). However, the poisoning of the Ti target could become predominant over time due to dynamic interactions between all components. If the Ti target poisoning becomes predominant, the formation of the TiO_*x*_‐CeO_*x*_ NPs will eventually terminate.

### 2.2 Formation of TiO_*x*_‐CeO_*x*_ Nanoparticles Without Additional Oxygen

**Figure 5:**
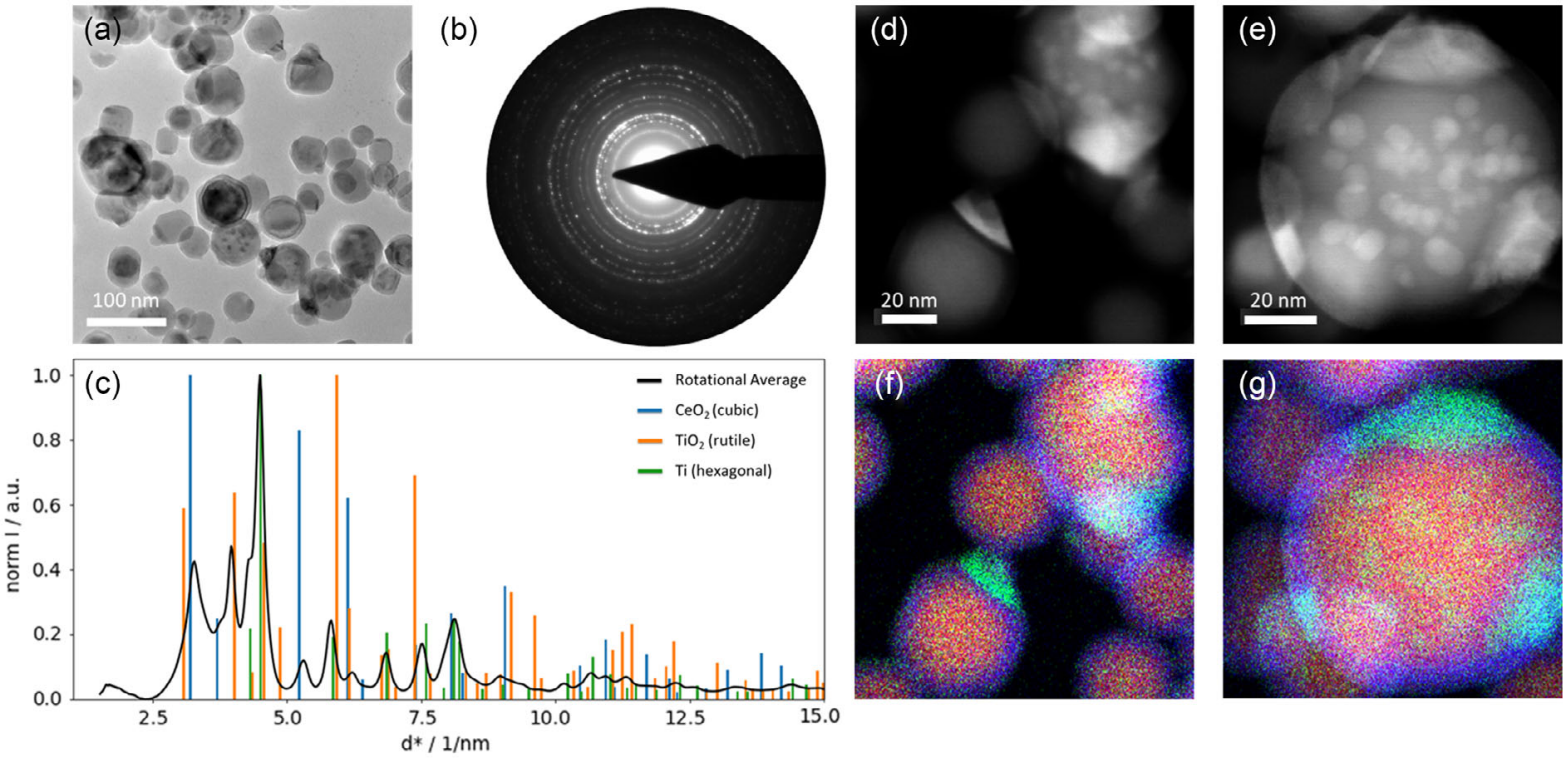
a) BF TEM image of particles deposited without the addition of oxygen. b) SAED pattern acquired from an area with several hundreds of particles. c) Rotational average of the SAED from (b) with references for CeO_2_ (ICSD 621 710),^[^ ^86^ ^]^ Ti (ICSD 168 830),^[^ ^92^ ^]^ and rutile phase TiO_2_ (ICSD 33 842).^[^ ^93^ ^]^ d,e) show ADFSTEM images of particles with core‐shell, multicore, and Janus‐type morphology. The related overlay EDX maps are given in f,g). The Ce elemental map is colored in green, Ti in red, and O in blue. The NPs were deposited from the segmented target with 0.25″ Ce inlet.

With the addition of a Ce inlet to the Ti target, particle formation was observed to be possible even without the addition of oxygen to the working gas. In depositions from the segmented target with the 0.25″ Ce inlet under these conditions, new features were observed in TEM. Next to Janus‐type and core‐shell particles, also multicore particles were synthesized. **Figure** **5**a shows a BF TEM image where the presence of these three morphology types can be seen. From the SAED pattern and the resulting rotational average (Figure 5b,c), crystallographic information on the particles can be extracted. Next to the cubic CeO_2_ that was also observed in depositions with additional oxygen before, non‐oxidized, metallic Ti and a small amount of rutile phase TiO_2_ are present in the sample. The EDX line scan in Figure S3, Supporting Information, supports the interpretation of metallic Ti being present in the core of the particle. Compared to the line scan from Figure S2, Supporting Information, the O‐K signal is significantly lower relative to Ti‐K and decreases toward the center of the particle. This indicates that the core contains very little to no oxygen. The presence of oxygen‐containing crystal phases in the sample hints toward the presence of residual O_2_ in the GAS during synthesis. The details of the expected particle formation mechanism without additional oxygen are discussed in later sections. The combined deposition of Ce and Ti seems to facilitate the nucleation of Ti without additional oxygen admixture. The ADF STEM images and EDX maps at higher resolution clearly show the spatial separation into Ti‐rich and Ce‐rich segments of the particles. As for depositions with the addition of oxygen, the smaller “cap” segment of the Janus‐type particles is Ce‐rich, while the larger segment of the particle is Ti‐rich (Figure 5d,f). In the multicore particle presented in Figure 5e,g, the major body of the particle is Ti‐rich. Ce is present not only in the “cap” segments of the particles but also in multiple small cores surrounded by the Ti‐rich matrix. From the combination of SAED and EDX results, it can be presumed that the metallic Ti is mostly present in the center of the particles, while the rutile phase TiO_2_ forms in the oxidized shell. As discussed already for the case with oxygen addition, it cannot be excluded that further crystal phases are present since their reflections could be overshadowed by high intensity reflections of the dominant phases. The differences in morphology as well as in crystal structure upon reduction of available oxygen during the particle formation process provide valuable information on the nucleation and growth process, which will be combined with the following XPS results. Afterward, the combined conclusions from TEM and XPS will be used to propose another particle formation mechanism for the case of decreased abundance of reactive gas.

**Figure 6:**
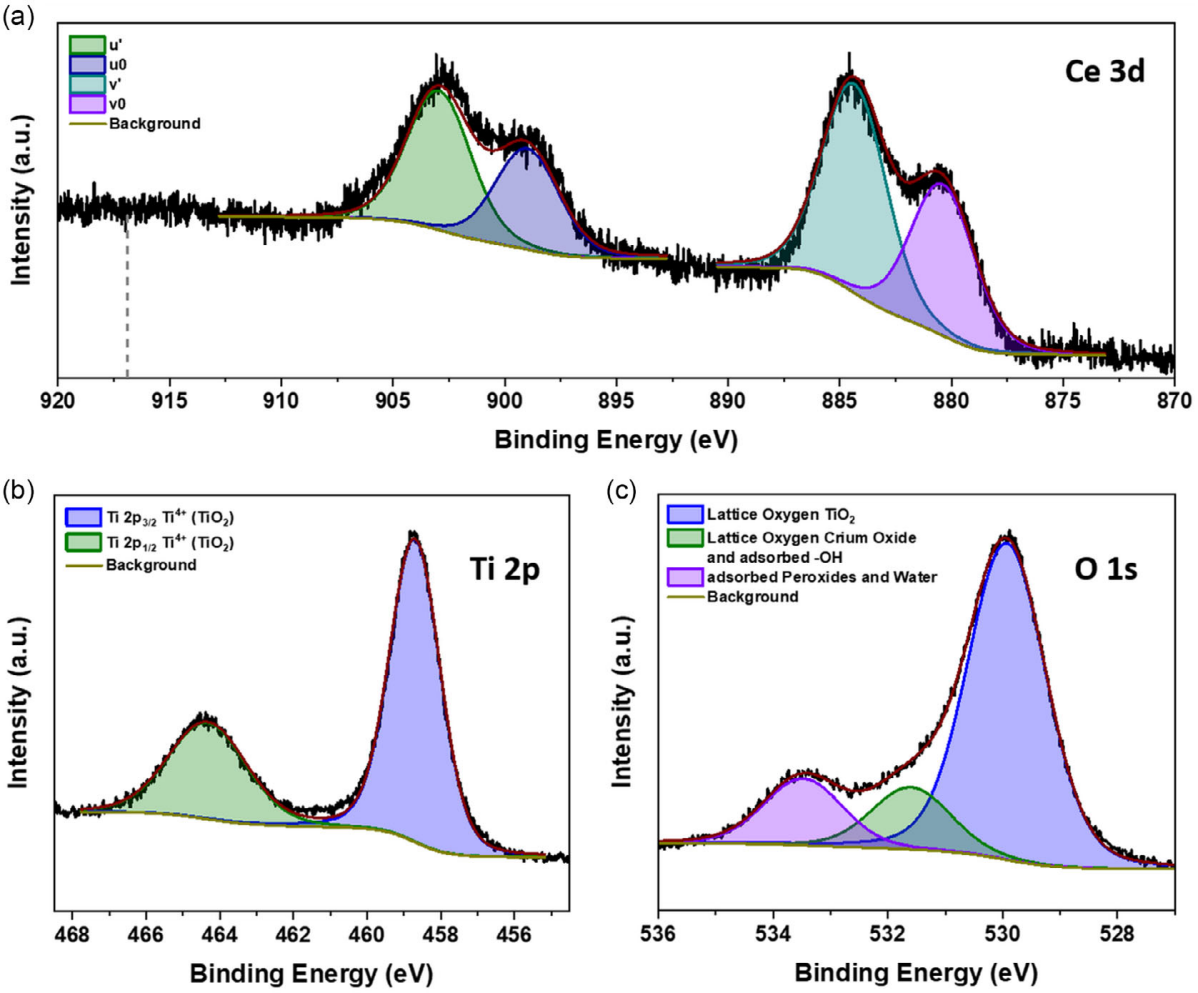
XPS core‐level spectrum of a sample produced without external O_2_ with respective fitting functions and background: a) Ce 3*d* with indicated binding energy position at 916.9 eV, b) Ti 2*p*, and c) O 1*s*.

We performed XPS on the NPs produced without external oxygen to analyze their surface chemistry. **Figure** **6**a shows the corresponding Ce 3*d* spectrum. Similar to the analysis on the samples with external oxygen, we fit multiples *u*′, *u*_0_ for Ce 3*d*_3/2_ and *v*′, *v*_0_ for Ce 3*d*_5/2_ with the same constraints we used for the Ce 3*d* peak in Figure 4. Again, the spectrum strongly suggests Ce^3+^ at the NP's surface.^[^ ^64^, ^65^, ^66^, ^67^ ^]^ Figure 6b displays the Ti 2*p* spectrum. It shows, comparable to the sample produced with external oxygen, two characteristic spin‐orbit components. Similar to the XPS results from Figure 4, we set the binding energy position of the Ti 2*p*_3/2_ component to a literature value of Ti^4+^, which is at 458.7 eV.^[^ ^76^ ^]^

To further clarify the surface oxidation states, we examine the O 1*s* spectrum. Figure 6c shows the O 1*s* spectrum with components for peroxides and adsorbed water, adsorbed –OH groups overlapping with lattice oxygen in Ce_2_O_3_, and lattice oxygen in TiO_2_. The main component at a binding energy of 529.9 eV lies within the reference values for lattice oxygen in TiO_2_.^[^ ^69^, ^70^ ^]^ The difference Δ_O1*s*–Ti 2*p*_ results in 71.2 eV, which is the same as the internal Ti^4+^ indicator Δ_O1*s*–Ti 2*p*,ref_ ≈ 71.2 eV defined with respect to Figure 4. Therefore, the Ti at the surface is most likely in the Ti^4+^ state. The second component at 531.6 eV corresponds to an overlap of lattice oxygen in Ce_2_O_3_^[^ ^66^, ^75^ ^]^ and adsorbed –OH.^[^ ^29^, ^75^ ^]^ We associate the third component at 533.5 eV to adsorbed oxygen in the form of peroxides and water.^[^ ^77^, ^78^ ^]^ In contrast to the NPs produced with external oxygen, this peroxide subpeak is not the major component here. Instead, the major component is the lattice oxygen in TiO_2_.

In summary, the spectra in Figure 6 indicate the Ti oxidation state of Ti^4+^ at the surface. The Ce is most likely in the Ce^3+^ state. Considering that for the production of the corresponding NPs, the plasma in the sputtering process was not supplied with external oxygen, complete oxidation to Ti^4+^ seems unlikely at first glance. However, surface oxidation after exposure to the atmosphere may be due to the strong oxygen affinity of Ti,^[^ ^85^ ^]^ which could be a reason why we observed Ti^4+^. This motivates a study where the vacuum between the NP production and the characterization is excluded as much as possible with the goal of analyzing the NP chemistry in the as‐deposited state to gain more information on the particle formation mechanism. Therefore, we produced a set of NP samples without the addition of oxygen as reactive gas and used vacuum transfer holders for TEM and XPS investigations to minimize the exposure to the ambient air.

### 2.3 Characterization Without Contact to Ambient Oxygen

**Figure 7:**
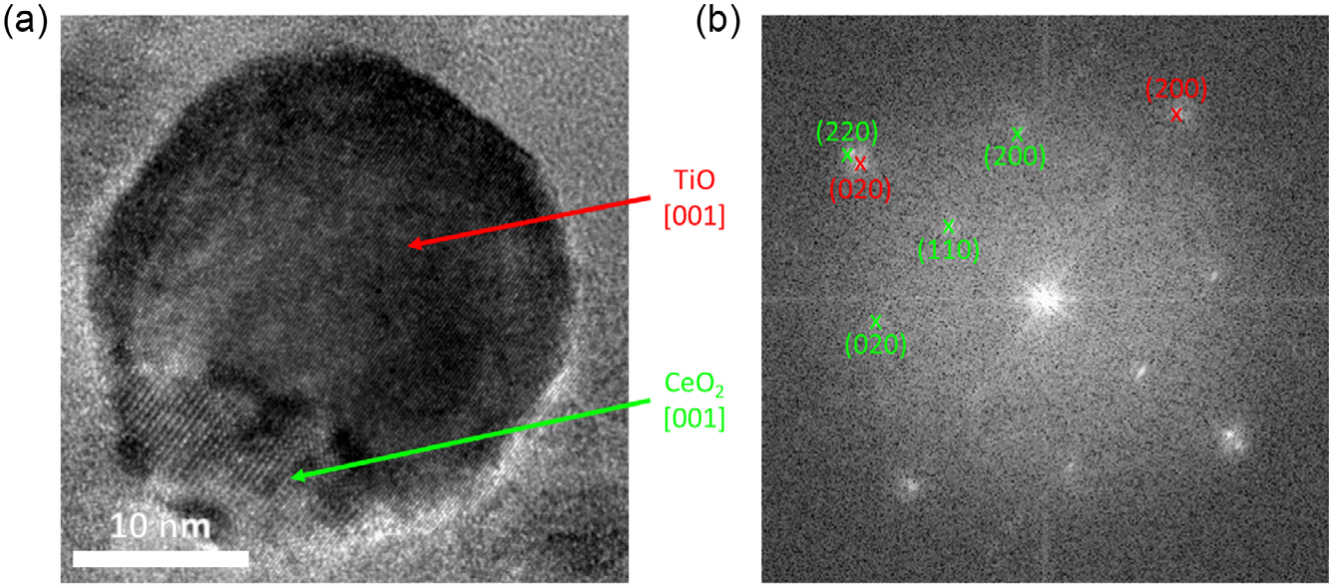
a) HRTEM micrograph of a single Janus‐type NP that was transferred to the TEM without contact to ambient oxygen. b) FFT of the NP depicted in (a). The reflections corresponding to cubic TiO and CeO_2_ with zone axis [001] are highlighted in red and green, respectively.

**Figure** **7** shows the high‐resolution TEM (HRTEM) micrograph and the corresponding fast fourier transform (FFT) of an NP produced without external oxygen. Additionally, the NP was analyzed without exposure to ambient oxygen after the sample production. On the HRTEM micrograph, two crystallographically different regions can be observed. From the inverse FFTs of single peaks of the FFT, these two regions can be identified as fcc TiO and fcc CeO_2_, both in [001] orientation. The two crystallites are rotated along the zone axis by about 45° with respect to each other. This creates an interface with well‐matching lattice parameters ((220) of CeO_2_ and (020) of TiO at almost identical positions in the FFT). The phases and orientations were identified relating to references ICSD 621 710^[^ ^86^ ^]^ and ICSD 77 692^[^ ^87^ ^]^ for CeO_2_ and TiO, respectively. The epitactic relationship between the two phases hints toward crystal growth of one component on top of the other using the first component as a crystal seed. The appearance of the forbidden (110) peak of CeO_2_ might result from the high defect level indicated by the significant Ce^3+^ concentration measured in XPS (presented in the following section).

Additionally, in the sample without contact to ambient oxygen, no particles with core‐shell morphology were observed. Such particles were found in the identical sample after two months of exposure to ambient air. This suggests that the amorphous oxidized shell forms on the surface of the initial particles upon exposure to oxygen in ambient air. Exemplary particles are presented in Figure S4, Supporting Information.

**Figure 8:**
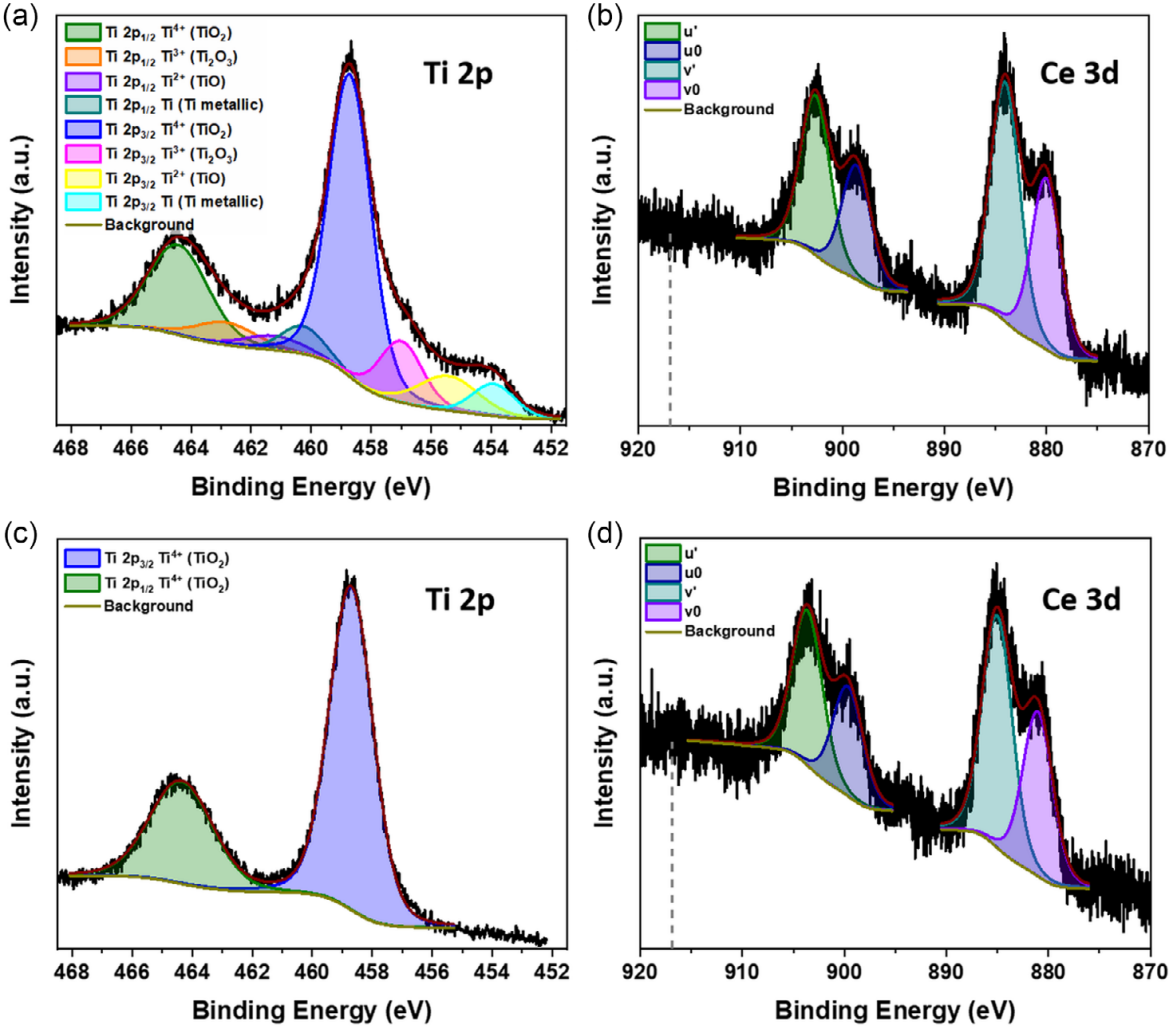
XPS core‐level spectra recorded directly after the vacuum transfer of the NP sample produced without external oxygen: a) Ti 2*p*, b) Ce 3*d*, and corresponding spectra recorded after two months of sample exposure to the atmosphere: c) Ti 2*p* and d) Ce 3*d*. All spectra include respective fitting functions and backgrounds. The Ce 3*d* spectra include indication lines at the binding energy position of 916.9 eV.

To investigate how the surface chemistry changes between the as‐deposited state and the state after significant amount of atmosphere exposure, we conducted two XPS measurements. The first XPS measurement included a sample vacuum transfer from the GAS sputter machine to the XPS system allowing for a minimum exposure to atmospheric gases. Afterward, we exposed the sample for two months to the atmosphere and performed a second XPS measurement. **Figure** **8**a,b shows the corresponding Ti 2*p* and Ce 3*d* spectra before the atmosphere exposure and Figure 8c,d shows the corresponding Ti 2*p* and Ce 3*d* spectra after atmosphere exposure.

The Ti 2*p* spectrum of the sample transferred under vacuum exhibits several subpeaks next to the two spin‐orbit split Ti 2*p*_1/2_ and Ti 2*p*_3/2_ main peaks. The main peaks with binding energy positions of 464.4 eV (Ti 2*p*_1/2_) and the set component at 458.7 eV (Ti 2*p*_3/2_) associate with the Ti^4+^ oxidation state. The evaluation of the difference Δ_O1*s*–Ti 2*p*_ calculated with the corresponding O 1*s* peak from Figure S5a, Supporting Information, yields 71.2 eV, which matches the internal Ti^4+^ reference Δ_O1*s*–Ti 2*p*,ref_ ≈ 71.2 eV. The additional subpeaks in the Ti 2*p* spectrum appear at lower binding energies and therefore correspond to lower oxidation states.^[^ ^88^, ^89^, ^90^, ^91^ ^]^ In descending binding energy from the respective Ti ^4+^ main peak, these lower oxidation state subpeaks associate with Ti^3+^, Ti^2+^, and Ti^0^.^[^ ^89^ ^]^ Therefore, the Ti 2*p* spectrum directly after the vacuum transfer indicates that the Ti of the as‐deposited NPs surface is not completely oxidized. After two months of atmosphere exposure, the Ti 2*p* spectrum displays only two spin‐orbit components at binding energies of 464.4 eV (Ti 2*p*_1/2_) and the set energy of 458.7 eV (Ti 2*p*_3/2_). Here, the difference Δ_O1*s*–Ti 2*p*_ calculated with the corresponding O 1*s* peak from Figure S5b, Supporting Information, is at 71.5 eV and thereby very close to Δ_O1*s*–Ti 2*p*,ref_ ≈ 71.2 eV. This indicates that the Ti at the NP surfaces reacts from a partially oxidized state to a fully oxidized Ti^4+^ state after the as‐deposited NPs get in contact with the atmosphere.

In contrast, the Ce 3*d* spectra before and after the atmosphere exposure do not show great differences. Both spectra display two main spin‐orbit components Ce 3*d*_3/2_ at high binding energies and Ce 3*d*_5/2_ at low binding energies. Those components split up into the subpeaks *u*′ and *v*′ as well as *u*_0_ and *v*_0_, which correspond to different final states that we discussed with respect to Figure 4 and 6. Additionally, the spectra do not show the Ce^4+^ characteristic *u*‴ component at 916.9 eV.^[^ ^64^ ^]^ Therefore, both spectra strongly suggest the presence of Ce^3+^ at the NP's surface. This implies that the Ce of the as‐deposited NP surface is in the Ce^3+^ state, which does not change after two months of atmosphere exposure. The Ce^3+^ is most likely stable at the surface.

The corresponding O 1*s* spectra from Figure S5, Supporting Information, show that the relative amount of adsorbed oxygen in the form of peroxides and water increased after the two‐month atmosphere exposure. However, similar to the sample we produced without external O_2_, adsorbed peroxides and water are not a major part of the O 1*s* peak. That is again a difference to the sample produced with external O_2_, where the O 1*s* peak is dominated by the peroxides and water component. These results match the corresponding TEM results. On the sample produced without external oxygen and analyzed without exposure to ambient oxygen, we observed a significant number of particles without an amorphous shell. In the discussion with respect to Figure 4, we associated a dominant peroxide subpeak in the O 1*s* spectrum with the oxygen‐rich surfaces of the core‐shell particles produced with external oxygen. When we supply no external oxygen, this core‐shell geometry and the relative number of adsorbed peroxides decrease.

**Figure 9:**
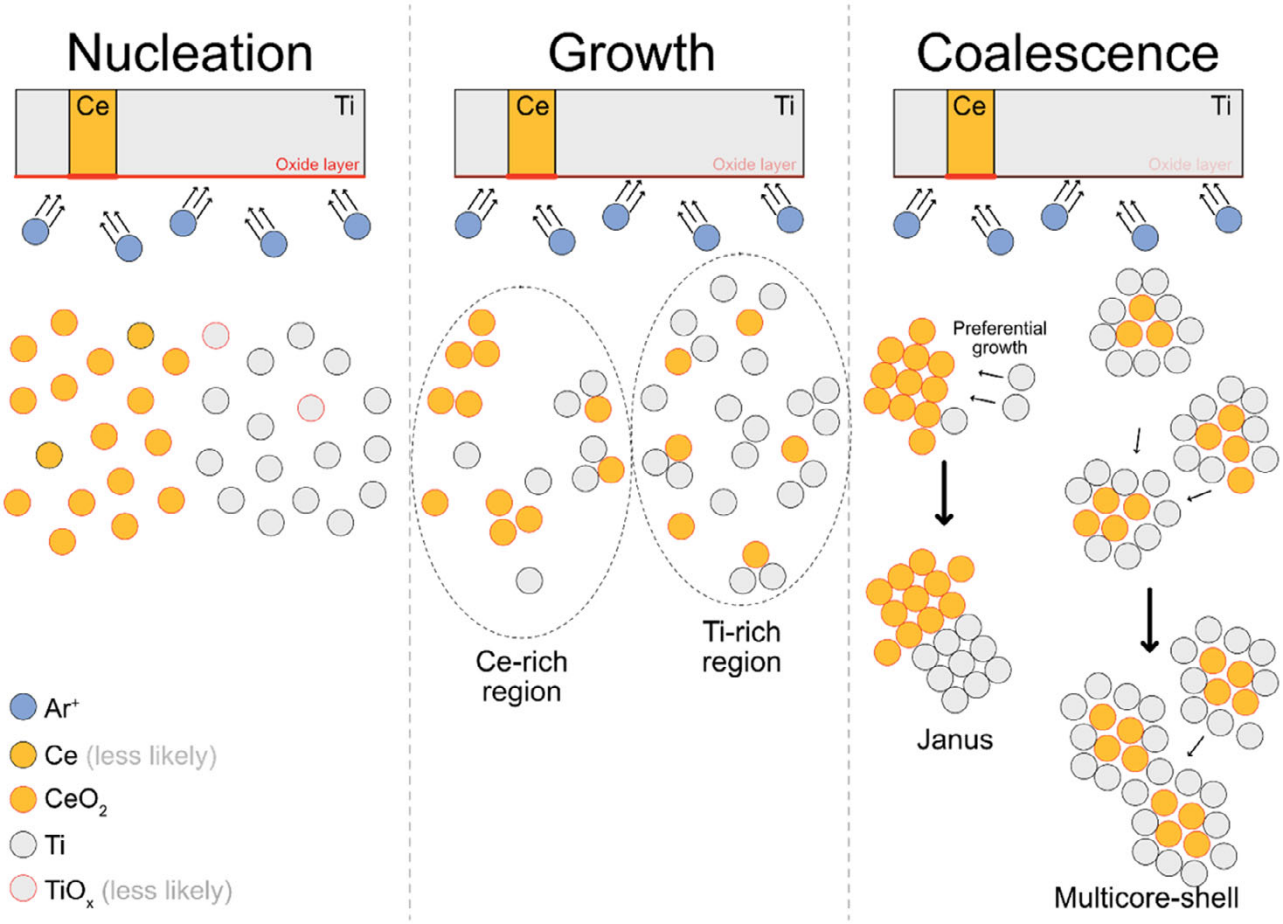
Schematic illustration of the formation mechanism of TiO_*x*_‐CeO_*x*_ nanoparticles without external O_2_ supply.

Based on the obtained TEM and XPS results, we propose a model for the formation of defective TiO_2_‐CeO_2_ NPs (mostly multicore‐shell and Janus) from the composite Ti‐Ce target without an external O_2_ supply as schematically shown in **Figure** **9**. In general, the formation of TiO_2_‐CeO_2_ NPs in a GAS involves several distinct processes. In such scenarios, the behavior and interaction of Ti, Ce, and available O_2_ under vacuum conditions play a crucial role in determining the characteristics and composition of the resulting defective TiO_2_‐CeO_2_ NPs. As already known, there is always a residual amount of O_2_ adsorbed in the deposition chamber, including the GAS walls even under high vacuum conditions (e.g., 10^−7^ mbar). Based on this scenario, the limited availability of O_2_ molecules adsorbed on the cluster wall can lead to a brief period of metal oxide deposition, such as TiO_2_, until all the adsorbed O_2_ is consumed.^[^ ^48^ ^]^ When using a Ti‐Ce segmented target, the difference in affinity for O_2_ between Ti and Ce becomes significant. Ce has a higher affinity for O_2_, which easily leads to the formation of an oxide layer on the metallic Ce target. This process depletes the available O_2_ in the deposition chamber, hindering the formation of a poisoning layer on the metallic Ti target. We have assumed that in the absence of external O_2_, this less poisoning layer becomes a more critical factor for the continuous and stable deposition of TiO_2_‐CeO_2_ NPs (the deposition rate of TiO_*x*_‐CeO_*x*_ NPs is around 1.88–2.73 Hz for >60 min).

In the GAS process, the excited Ar^+^ ions then bombard the target surface, removing Ce and Ti atoms. Initially, a CeO_2_ layer forms on the metallic Ce target surface due to adsorbed O_2_ in the deposition chamber. This CeO_2_ layer can be easily removed by Ar^+^ bombardment, forming initial CeO_2_ nuclei in the chamber. Similarly, the first Ti atoms can be extracted from the metallic Ti target surface, which has a very thin oxide layer. Due to the asymmetric composite target configuration, two distinct regions may form based on the concentration of Ce and Ti atoms: In the Ti‐rich region, Ti atoms form over the early nucleated CeO_2_ clusters. Here, CeO_2_ clusters do not grow significantly because the abundance of Ti atoms disturbs the formation of additional CeO_2_ clusters. This process can lead to the formation of multicore‐shell structures. However, in the Ce‐rich region, CeO_2_ clusters can easily form and grow larger due to the comparably high concentration of Ce species. In this region, Ti atoms attach in specific directions on the CeO_2_ clusters (preferential growth), leading to the formation of Janus TiO_2_‐CeO_2_ NPs.

**Figure 10:**
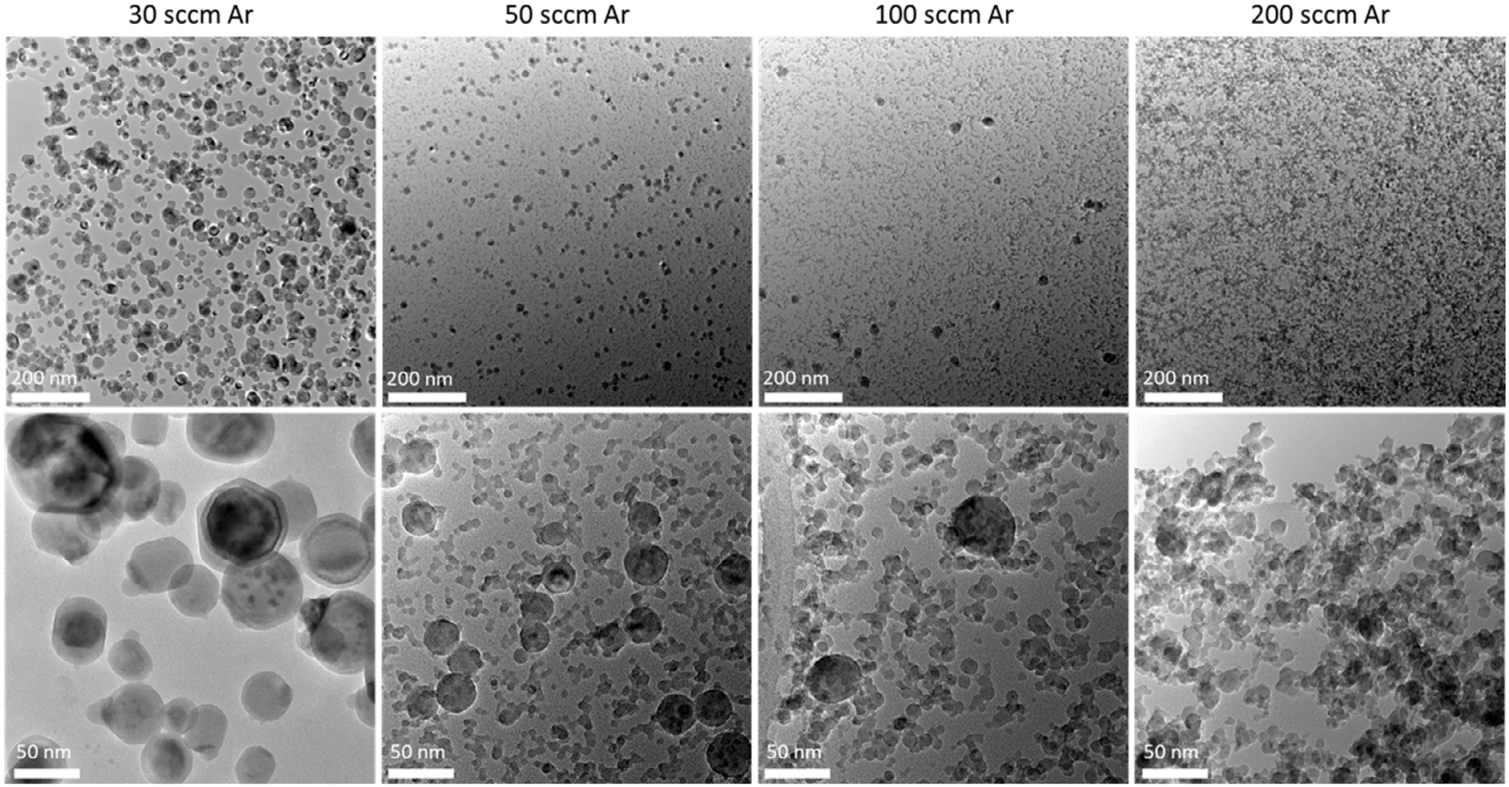
BF TEM images of particles synthesized from the segmented target with 0.25″ Ce inlet at different Ar flow rates deposited directly on the TEM grid. The amount of larger particles reduces with increasing Ar flow rates.

As expected, any changes in the dynamic of the deposition process might drastically affect the formation of the particles. The pressure in the deposition chamber could be one of the dominant factors in the particle size and distribution. Therefore, we gradually change the pressure in the deposition chamber by changing the Argon flow rate (30–200 sccm). **Figure** **10** shows the adjusted Ar flow rate has a significant influence on the particle size distribution. The estimated particle size distribution is depicted in Figure S6, Supporting Information. From the image as well as from the size distribution histograms, significant differences in particle diameters between samples deposited at different Ar flows can be observed. At 30 sccm Ar, most particles have diameters of about 40 nm, while the size distribution is comparably broad and ranges from less than 5 nm to more than 100 nm. When the Ar flow is increased to 50 sccm, the particle size distribution undergoes drastic changes. The majority of particles show diameters between 5 nm and 15 nm. Next to these small particles, a low but still significant number of larger particles with diameters between 25 and 40 nm can be found. An increase in Ar flow to 100 sccm leads to a further reduction in the number of larger particles. Out of 500 measured particles, only 3 have diameters between 45 and 50 nm, while all other particles have diameters below 20 nm with a maximum of about 10 nm. An Ar flow of 200 sccm broadens the particle size distribution in comparison to 100 sccm. The distribution still has its maximum of about 10 nm but spreads to slightly larger particle sizes of up to 35 nm. Here, the group of “larger” particles has vanished.

## 3 Conclusion

In the presented study, we propose a novel approach for the fabrication of MMO NPs in a single‐step vacuum‐based synthesis process employing a GAS‐based deposition system. In the case of NPs from TiO_*x*_ and CeO_*x*_, the formation of a variety of morphologies (core‐shell, Janus‐type, and multicore) was observed. Here, the amount of O_2_ in the system plays a crucial role, not only influencing the chemistry of the particle formation process but also impacting the sputtering of Ce and Ti via the formation of a poisoning layer. Next to the interesting morphological features of the synthesized particles, the surface of the produced material was found to be highly defective, containing high amounts of Ce^3+^, which is known to be beneficial in a wide range of applications including catalysis, photocatalysis, and supercapacitors, as well as a significant amount of Ti^3+^, Ti^2+^, and Ti^0^. While the Ti surface completely oxidizes to Ti^4+^ when exposed to ambient O_2_, the Ce^3+^ at the surface was found to be stable. Changes in the particle size and chemical composition are proven to be feasible via the adjustment of the reactive gas flow and the initial target composition. Regardless of the complex mechanisms influencing the resulting particles in MMO NP synthesis by GAS, which require further research to be fully understood, we believe the technique to be a high potential versatile tool to fabricate high‐purity composite NPs from a wide range of materials with a wide range of different properties.

## 4 Experimental Section

4.1

4.1.1

##### Fabrication of TiO_x_‐CeO_x_ Nanoparticles

We deposited TiO_*x*_‐CeO_*x*_ NPs on silicon (Si) and quartz glass wafers as well as various TEM grids with a custom‐built 2‐inch magnetron (Thin Films Consulting; Ionix IX2U) GAS. At the magnetron, we inserted different segmented Ti/Ce targets. For the fabrication of the targets, we combined 2‐inch Ti targets (99.995% purity, Kurt J. Lesker) with 1″ (99.995% purity, Kurt J. Lesker), 0.5″, and 0.25″ sized (99.9% purity, Testbourne) Ce inlets. The cluster source of this study has a length of 177 mm and a diameter of 98 mm. It terminates by a conical lid with a spherical orifice of 2.0 mm diameter, which links the GAS to the main deposition chamber. To establish a vacuum of ≈4 × 10^−6^ mbar base pressure, we used a turbo molecular pump (Pfeiffer Vacuum, HiPace 400) supported by a Roots pump (Pfeiffer Vacuum, ACP 15). For the TiO_2_‐CeO_2_ NP deposition, the magnetron of the cluster source was operated in DC mode (power supply, BeamTec, MPS1500P). We utilized argon (Ar) as an inert sputter gas. A precise mass‐flow controller (MKS Instruments Deutschland GmbH, 1179BX22CM1BV with 200 sccm range) regulated its flow. As reactive gas, we introduced oxygen (O_2_) with another mass‐flow system (APEX/ALICAT, AX‐MC‐1SCCM) upon operation. We preceded each deposition by a thorough cleaning of the target. Here, the power supply (BeamTec, MPS1500P) was operated at low power (30 W DC), and we set the Ar flow to 5 sccm, resulting in ≈53 Pa of source pressure. Upon cleaning, we increased the magnetron power in 5‐min steps from 30 to 50 to 70 W, successively. The cleaning ended when the discharge voltage of the target was reduced to ≈150 V after at least 8 min of cleaning. Afterward, we followed with a conditioning phase starting with 30 sccm of pure Ar flow at a power of 50 W. For selected depositions, we increased the power to a power 70 W, and we adjusted a small O_2_ flow of 0.015 sccm for the depositions with external O_2_. With a quartz crystal microbalance (QCM, Kurt J. Lesker) connected to a rate monitor (STM‐100/MF, Sycon Instruments Pvt. Ltd.), we observed the relative deposition rate until the deposition rate and the discharge voltage remained constant for the desired flow and power settings. For the deposition, we retracted the QCM, and the discharge voltage was closely monitored. A typical deposition included 10–30 min of deposition time, tailored to reach the optimum amount of deposited material for the respective analysis technique at the given deposition rate.

##### Characterization: Transmission Electron Microscopy

Particles that were originally deposited on Si or quartz wafer pieces were scratched off the substrate surface with a scalpel and transferred to a copper (Cu) TEM grid with a lacey carbon support (Plano GmbH). Alternatively, the Si substrates were ultrasonicated in butanol, and the dispersion was drop‐casted onto a Cu lacey TEM grid. Additional TEM samples were prepared by depositing the particles directly on TEM grids. For transfer from the deposition chamber to the TEM vacuum without contact to ambient oxygen, a custom‐made vacuum transfer holder for the deposition chamber was designed. After deposition, the particles deposited directly on a TEM grid were inserted inside the sealed holder into a glove box with an Ar atmosphere. In this inert surrounding, the grid was mounted into a TEM vacuum transfer holder (Gatan model 648). The Ar protection in the TEM holder was maintained to insert the sample into the TEM and released in the pre‐chamber after evacuation.

BF imaging, HRTEM, and SAED were performed in a FEI Tecnai F30 STwin *G* ^2^ with 300 kV acceleration voltage. The instrument is equipped with a Si/Li detector (EDAX System) that was used to acquire averaged EDX spectra.

ADF imaging and EDX mapping were performed using a probe‐corrected STEM (Jeol JEM‐ARM200F Neoarm) at 200 kV acceleration voltage with two windowless silicon drift detectors to acquire the EDX signal. The elemental maps were created from the Ti‐K, Ce‐L, and O‐K lines, respectively.

##### Characterization: X‐ray Photoelectron Spectroscopy

The surface elemental composition and chemical states were analyzed with XPS. The XPS UHV system, manufactured by PREVAC Sp. z o. o. with a 300 W Al‐anode, was used to record the spectra. Wide scans were recorded with three iterations and a pass energy of 200 eV. High‐resolution scans were recorded with 20 iterations and a pass energy of 50 eV. For further processing and analysis of the obtained XPS spectra, the CasaXPS (version 2.3.25) software was utilized. First, the Shirley algorithm was applied to quantify the background of each spectrum. Additionally, all relevant high‐resolution peaks were fitted using the Lorentzian (LA) line shape model, which is a convolution of Gaussian and Lorentzian functions. The charge correction procedure involved fitting the Ti 2*p*_3/2_ main peak and setting the peak position of the fit to the reference value of 458.7 eV, which is a reference value for Ti^4+^.^[^ ^76^ ^]^ Subsequently, all relevant spectra were adjusted accordingly, with the same amount of energy shift applied.

We transferred a selected sample produced without external oxygen from the GAS vacuum chamber with a custom‐made vacuum transfer holder via a KF flange to the XPS system. During this process, the transfer holder should keep the sample in a high vacuum without exposure to the atmosphere. Upon transfer, the KF flange leaked, and atmospheric gases came into contact with the sample for about 30 s. However, we still present the corresponding data since it shows even with that short breakthrough, a partially reduced titanium oxide at the surface which we associate with the as‐deposited state.

## Conflict of Interest

The authors declare no conflict of interest.

## Author Contributions

**Marie Elis**: Conceptualization (supporting); Formal analysis (equal); Investigation (equal); Methodology (equal); Validation (supporting); Visualization (equal); Writing—original draft (equal); Writing—review & editing (equal). **Tim Tjardts**: Conceptualization (supporting); Formal analysis (equal); Investigation (equal); Methodology (equal); Validation (supporting); Visualization (equal); Writing—original draft (equal); Writing—review & editing (equal). **Josiah Ngenev Shondo**: Formal analysis (supporting); Investigation (equal); Writing—original draft (supporting); Writing—review & editing (supporting). **Ainura Aliyeva**: Formal analysis (supporting); Investigation (supporting); Writing—original draft (supporting). **Alexander Vahl**: Conceptualization (equal); Formal analysis (supporting); Investigation (supporting); Visualization (supporting); Writing—original draft (supporting); Writing—review & editing (supporting). **Ulrich Schürmann**: Conceptualization (equal); Formal analysis (supporting); Investigation (supporting); Supervision (supporting); Writing—original draft (supporting); Writing—review & editing (supporting). **Thomas Strunskus**: Formal analysis (supporting); Validation (supporting); Writing—original draft (supporting); Writing—review & editing (supporting). **Franz Faupel**: Conceptualization (supporting); Funding acquisition (equal); Project administration (equal); Resources (equal); Supervision (equal); Writing—original draft (supporting); Writing—review & editing (supporting). **Cenk Aktas**: Conceptualization (equal); Funding acquisition (lead); Project administration (lead); Resources (equal); Supervision (equal); Writing—original draft (supporting); Writing—review & editing (supporting). **Lorenz Kienle**: Funding acquisition (lead); Project administration (lead); Resources (equal); Supervision (equal); Writing—original draft (supporting); Writing—review & editing (supporting). **Salih Veziroglu**: Conceptualization (lead); Formal analysis (equal); Investigation (equal); Project administration (equal); Resources (equal); Supervision (equal); Visualization (equal); Writing—original draft (equal); Writing—review & editing (equal). **Marie Elis** and **Tim Tjardts** contributed equally to this work.

## Supporting information

Supplementary Material

## Data Availability

The data that support the findings of this study are available from the corresponding author upon reasonable request.
