# Supplementary material for: A New Approach to Single‐Step Fabrication of TiO x ‐CeO x Nanoparticles
Source: Small Sci. 2024 Sep 9;4(11):2400305. doi: 10.1002/smsc.202400305 (PMC11935049; doi:10.1002/smsc.202400305)
Supplement: Supplementary file 1 — Supplementary Material [file SMSC-4-2400305-s001.pdf]

Supporting Information

A New Approach to Single Step Fabrication of TiO_x-CeO_x Nanoparticles

Marie Elis,[†] Tim Tjardts,[†] Josiah Ngenev Shondo, Ainura Aliyeva, Alexander Vahl, Ulrich Schürmann, Thomas Strunskus, Franz Faupel, Cenk Aktas, Lorenz Kienle, Salih Veziroglu**

[†] These authors contributed equally to this work.

Marie Elis, Ulrich Schürmann, Lorenz Kienle
Chair for Synthesis and Real Structure, Department of Materials Science, Faculty of Engineering, Kiel University, Kaiserstraße 2, 24143 Kiel, Germany
E-mail: lk@tf.uni-kiel.de

Tim Tjardts, Josiah Ngenev Shondo, Ainura Aliyeva, Alexander Vahl, Thomas Strunskus, Franz Faupel, Cenk Aktas, Salih Veziroglu
Chair for Multicomponent Materials, Department of Materials Science, Faculty of Engineering, Kiel University, Kaiserstraße 2, 24143 Kiel, Germany
E-mail: sve@tf.uni-kiel.de

Alexander Vahl
Leibniz Institute for Plasma Science and Technology, Felix-Hausdorff-Str. 2, 17489 Greifswald, Germany

Alexander Vahl, Ulrich Schürmann, Thomas Strunskus, Franz Faupel, Lorenz Kienle, Salih Veziroglu
Kiel Nano, Surface and Interface Science KiNSIS, Kiel University, Christian Albrechts-Platz 4, 24118 Kiel, Germany

Cenk Aktas
Department of Orthodontics, University Hospital of Schleswig-Holstein (UKSH), Kiel University, Arnold-Heller-Straße 3, 24105 Kiel, Germany

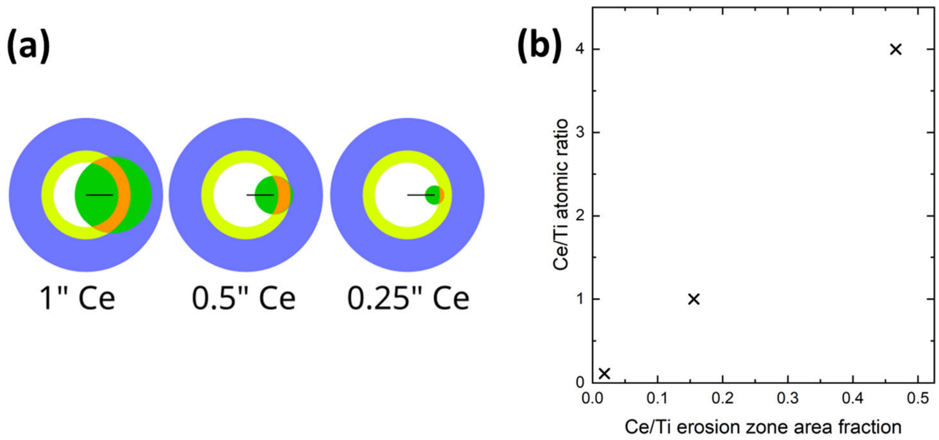

Figure S1. (a) Sketch of the Ce/Ti erosion zone area fraction using different size Ce inlet targets. (b) The relationship of Ce/Ti erosion zone area fraction to the composition of the TiO_x-CeO_x nanoparticles determined by EDX. Note that the erosion zone profile (i.e. a radius-dependent erosion profile depth) was not considered in this simplified model calculation.

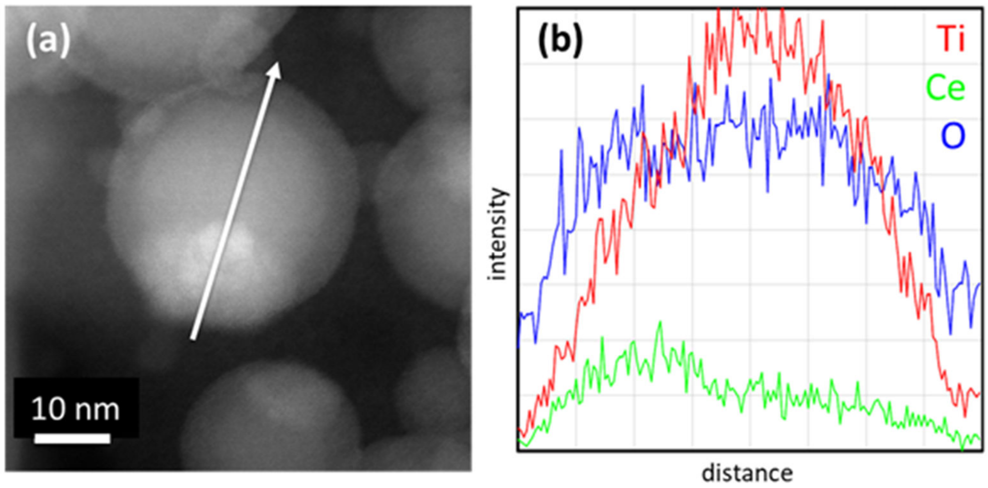

Figure S2. (a) ADF STEM image and (b) corresponding elemental signal from an EDX line scan along the white arrow marked in the image. The particles were deposited with the addition of 0.015 sccm O_2.

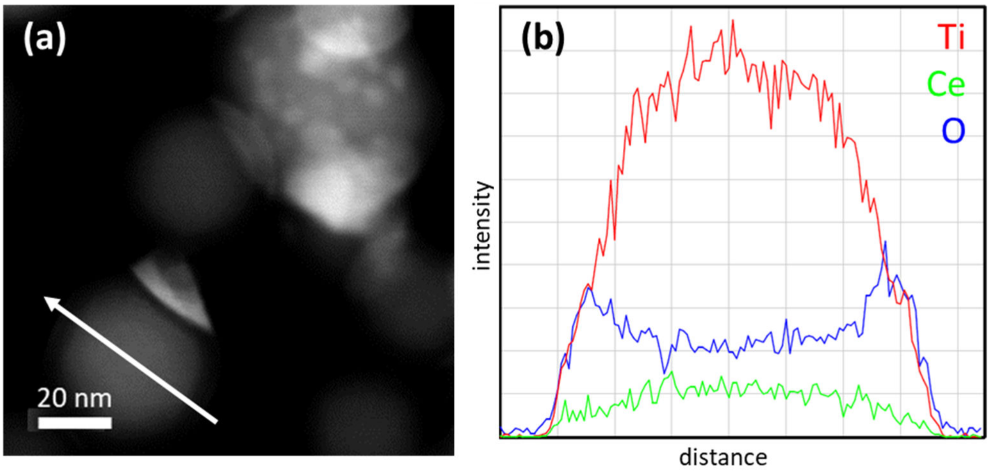

Figure S3. (a) ADF STEM image and (b) elemental EDX signal from a line scan along the white arrow in (a). The particles were deposited without addition of oxygen.

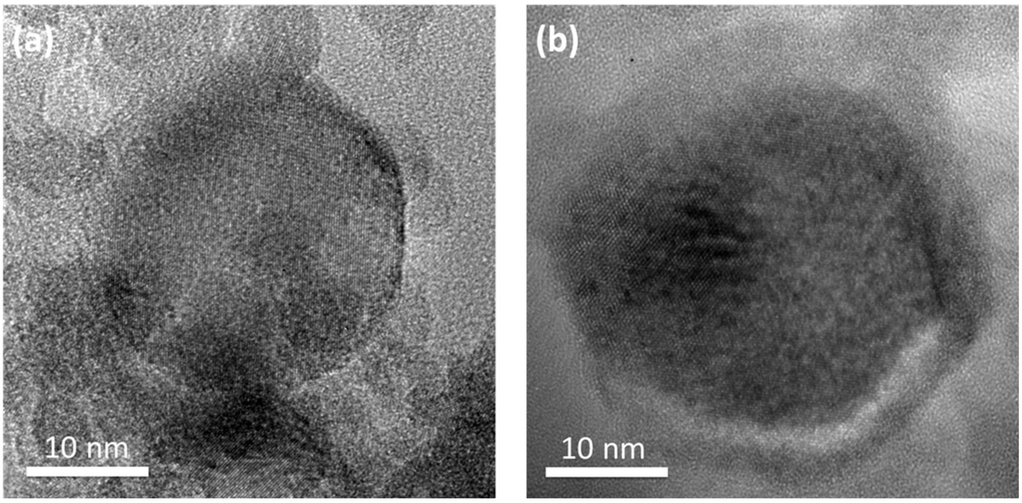

Figure S4. (a) HRTEM micrograph of an exemplary particle transferred to TEM without contact to ambient oxygen. (b) HRTEM micrograph of an exemplary particle with core-shell morphology after storage in atmosphere for 2 months.

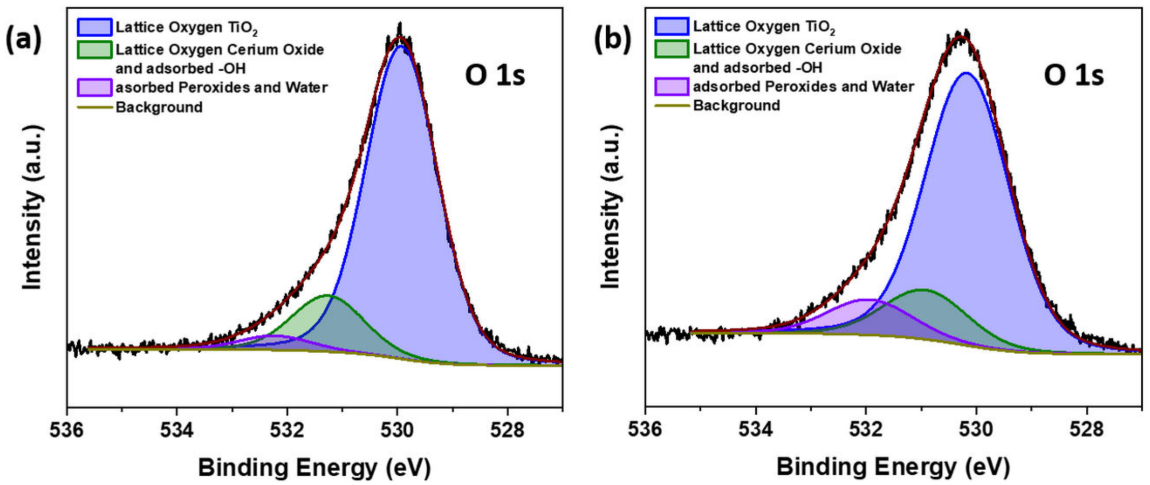

Figure S5. XPS O 1s core-level spectra: (a) recorded directly after the vacuum transfer, (b) recorded after 2 months of sample exposure to the atmosphere. All spectra include respective fitting functions and backgrounds.

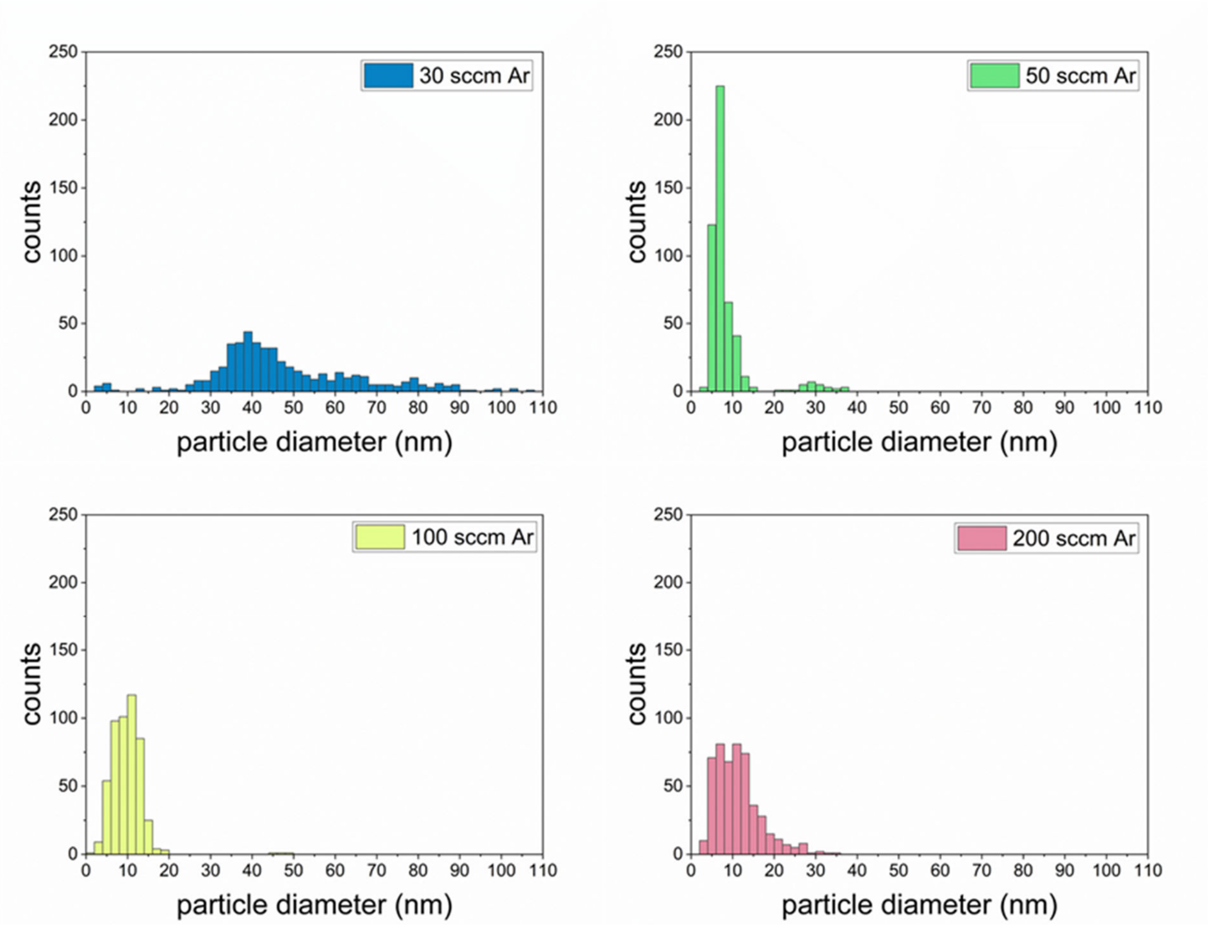

Figure S6. Distribution of particles diameters in samples prepared at different Ar flow rates. For each histogram, the diameters of 500 particles were measured via a customized measure features script in digital micrograph software.
